# Psychological Stress Management and Stress Reduction Strategies for Stroke Survivors: A Scoping Review

**DOI:** 10.1093/abm/kaac002

**Published:** 2022-06-11

**Authors:** Madeleine Hinwood, Marina Ilicic, Prajwal Gyawali, Kirsten Coupland, Murielle G Kluge, Angela Smith, Sue Bowden, Michael Nilsson, Frederick Rohan Walker

**Affiliations:** School of Medicine and Public Health, The University of Newcastle, Callaghan, NSW, Australia; Hunter Medical Research Institute, New Lambton Heights, NSW, Australia; Hunter Medical Research Institute, New Lambton Heights, NSW, Australia; School of Biomedical Sciences and Pharmacy, The University of Newcastle, Callaghan, NSW, Australia; Priority Research Centre for Stroke and Brain Injury, The University of Newcastle, Callaghan, NSW, Australia; School of Health and Wellbeing, Faculty of Health, Engineering and Sciences, University of Southern Queensland, Darling Heights, QLD, Australia; Hunter Medical Research Institute, New Lambton Heights, NSW, Australia; School of Biomedical Sciences and Pharmacy, The University of Newcastle, Callaghan, NSW, Australia; Priority Research Centre for Stroke and Brain Injury, The University of Newcastle, Callaghan, NSW, Australia; School of Biomedical Sciences and Pharmacy, The University of Newcastle, Callaghan, NSW, Australia; Centre for Advanced Training Systems, The University of Newcastle, Callaghan, NSW, Australia; HNE Health Libraries, Hunter New England Local Health District, New Lambton, NSW, Australia; Consumer Investigator, Moon River Turkey, Bathurst, NSW, Australia; Hunter Medical Research Institute, New Lambton Heights, NSW, Australia; Centre for Rehab Innovations, The University of Newcastle, Callaghan, NSW, Australia; LKC School of Medicine, Nanyang Technological University, Singapore; Hunter Medical Research Institute, New Lambton Heights, NSW, Australia; School of Biomedical Sciences and Pharmacy, The University of Newcastle, Callaghan, NSW, Australia; Priority Research Centre for Stroke and Brain Injury, The University of Newcastle, Callaghan, NSW, Australia; Centre for Advanced Training Systems, The University of Newcastle, Callaghan, NSW, Australia; Centre for Rehab Innovations, The University of Newcastle, Callaghan, NSW, Australia

**Keywords:** Stroke, Stress, Resilience, Stress intervention, Stress management, Depression

## Abstract

**Background:**

Stroke can be a life-changing event, with survivors frequently experiencing some level of disability, reduced independence, and an abrupt lifestyle change. Not surprisingly, many stroke survivors report elevated levels of stress during the recovery process, which has been associated with worse outcomes.

**Purpose:**

Given the multiple roles of stress in the etiology of stroke recovery outcomes, we aimed to scope the existing literature on stress management interventions that have been trialed in stroke survivors.

**Methods:**

We performed a database search for intervention studies conducted in stroke survivors which reported the effects on stress, resilience, or coping outcome. Medline (OVID), Embase (OVID), CINAHL (EBSCO), Cochrane Library, and PsycInfo (OVID) were searched from database inception until March 11, 2019, and updated on September 1, 2020.

**Results:**

Twenty-four studies met the inclusion criteria. There was significant variation in the range of trialed interventions, as well as the outcome measures used to assess stress. Overall, just over half (13/24) of the included studies reported a benefit in terms of stress reduction. Acceptability and feasibility were considered in 71% (17/24) and costs were considered in 17% (4/24) of studies. The management of stress was rarely linked to the prevention of symptoms of stress-related disorders. The overall evidence base of included studies is weak. However, an increase in the number of studies over time suggests a growing interest in this subject.

**Conclusions:**

Further research is required to identify optimum stress management interventions in stroke survivors, including whether the management of stress can ameliorate the negative impacts of stress on health.

## Teaser

In this review, we found that interventions to reduce stress in stroke survivors were highly variable, and although more than half reported a positive effect in reducing stress, the feasibility of deploying these interventions in practice was rarely considered.

## Introduction

Advances in the treatment of stroke, particularly the introduction of clot-busting drugs and clot retrieval technologies, have significantly reduced stroke mortality [1]. Improvements in diagnosis and rehabilitation have also improved stroke outcomes; however, many stroke survivors continue to experience poor health outcomes for their remaining lifespan. Stroke is one of the five leading global causes of disability-adjusted life years, and the number of years lost as a result of poor health or disability from cardiovascular disease (CVD), including stroke, is greater than the number of years lost to cardiovascular death globally [2]. This suggests an urgent need to identify new targets and interventions to improve quality of life (QoL) following stroke.

Psychosocial wellbeing after stroke has been relatively neglected compared with motor and other physical symptoms, which are often the primary focus of rehabilitation efforts. One emerging prognostic factor determining the quality of psychological and emotional recovery from stroke is stress [3, 4]. Stroke survivors report experiencing persistently high levels of stress, with greater levels of perceived stress poststroke associated with poorer long-term outcomes [5–10]. Several observational studies have consistently reported significant correlations between stress and worse stroke outcomes, including functional independence, psychological outcomes such as depression, and cognitive function. Likewise, greater resilience, which is defined as the capacity to withstand adversity and “bounce back” after a stressful event, is associated with better QoL poststroke [7, 11]. Further, stress is among the strongest proximal risk factors for depression and anxiety disorders, and the risk of these stress-related mental health disorders is significantly greater in stroke survivors compared with the general population [12]. In the 2 years immediately following stroke, the risk of depression for stroke survivors was around 25%, compared with 8% in a control group of people the same age [12]. Stroke survivors are also at increased risk of other stress-related disorders, including post-traumatic stress disorder (PTSD) and anxiety disorders [13, 14]. These psychological problems are independently associated with increased morbidity, mortality, and disability [15, 16]. In addition to a heightened risk of stress-related disorders, the recovery domains influenced by stress broadly contribute to worse QoL, reduced motivation and lower levels of self-reported wellbeing, which in turn may negatively impact participation in rehabilitation. This is likely to potentiate a positive feedback loop between heightened stress perception and poor participation in rehabilitation.

There is clear evidence that rehabilitation interventions can influence patient outcomes after stroke [15, 17]. Therefore, identifying and modifying alternative prognostic factors for recovery outcomes are of vital importance to improve trajectories for stroke survivors. Studies highlighting the association between stress and significant downstream effects such as emotional and cognitive problems and poor functional recovery suggest that managing stress may be beneficial to stroke survivors [8–10]. There is some evidence that stress management interventions in populations with other chronic illnesses, particularly cancer and CVD, can decrease symptoms of depression, and promote resilience [18, 19]. However, it is unclear which interventions to mitigate stress have been trialed for stroke survivors.

**Fig. 1.**
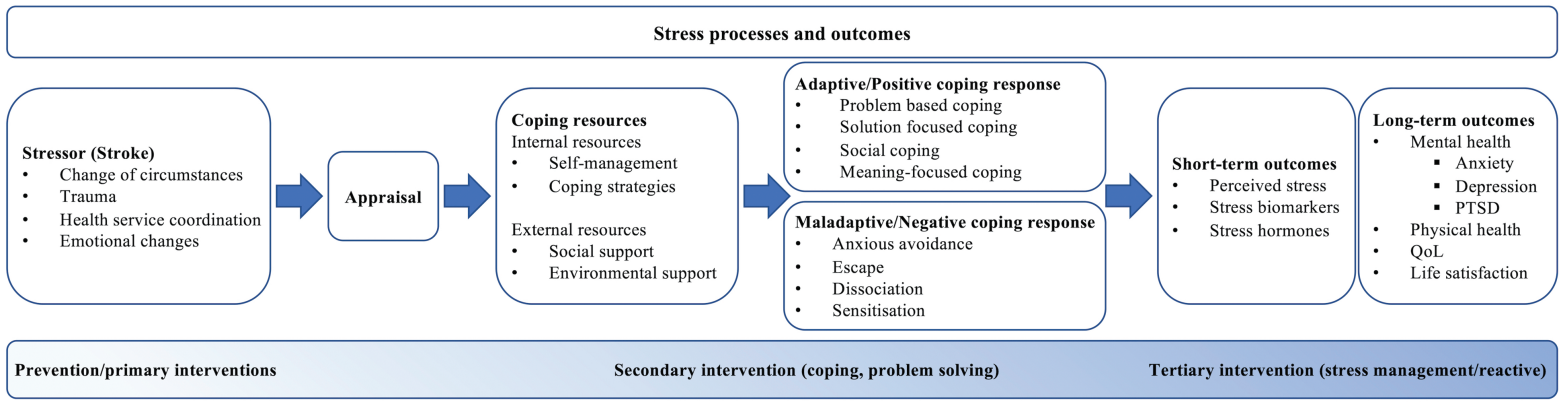
Conceptual framework of stress processes after stroke, and spectrum of interventions to manage or reduce stress (adapted from Folkman and Lazarus [20]).

Psychological stress is a complex phenomenon, and numerous theoretical models of stress have been proposed. There are also various terminologies in the literature to describe the evaluation of state stress. Variations in terminology connected to stress as a short- or long-term outcome may include stress, distress, depression, anxiety, coping, and QoL. The converse can also be identified; although stress exposure can have lasting negative impacts on psychological health and wellbeing, not all individuals will go on to develop these outcomes, and resilience scores are therefore also frequently examined [19]. In this study, we conceptualized psychological stress according to the stress, appraisal, and coping framework proposed by Lazarus and Folkman [20], adapted for the stroke setting in Fig. 1, where stress is a consequence of an individual’s appraisal of their environment, and their perceived ability to cope with a situation or incident. Therefore, depending on how it is appraised, a stressor may have differential short- and long-term effects upon an individual. Interventions to manage stress may be deployed at any point along a spectrum, with primary interventions primarily concerned with stressor reduction, secondary with stress management, and tertiary with remedial support or treatment of stress-related conditions. Here, we expected to find most interventions at the secondary (stress management) level, with outcomes primarily based on the effectiveness of the intervention in the short term (e.g., reduction in perceived stress or other stress marker; improvement in coping skills; or improvement in resilience), and on the ability of the intervention to prevent other downstream effects of stress, such as symptoms of anxiety and depression. Our framework guided the design of the research questions, literature search, identification of studies, and the collation of results.

Given that research into stress management strategies for stroke survivors is an emerging area of research, we collated the results of existing studies under several broad topic areas. In addition to mapping and assessing the reported effectiveness of interventions, we also aimed to collate information on the stress outcome measures used, whether long-term effects of stress were considered, and implementation outcomes (specifically acceptability, feasibility, and economic analyses).

Given the breadth of information to be accumulated, we decided to perform a scoping rather than a systematic review. Scoping reviews are designed to assess the coverage of a body of literature, and to identify gaps and map available evidence. Systematic reviews in health care tend to be more focused on confirming or refuting whether current practice is based on evidence, and to establish the quality of that evidence [21]. Our overall aim was to broadly map the body of literature concerning stress management interventions in stroke survivors. Further, we aimed to synthesize existing results and approaches around stress interventions in stroke survivors to guide the development and implementation of stress management in future studies, identify knowledge gaps, and clarify key concepts around the measurement of stress in stroke survivors. Therefore, as we were mapping a heterogeneous body of literature rather than synthesizing the best available research to answer a specific question, a scoping review methodology was considered most appropriate.

## Methods

This scoping review was conducted in accordance with the PRISMA extension for scoping reviews [22].

### Protocol

A protocol was published prior to conducting this review [23] based upon the framework proposed by Arksey and O’Malley [24] and updated by Levac et al. [25], and the methods outlined in the Joanna Briggs Institute Reviewers’ Manual [26]. The scoping review process is an iterative one, and the inclusion and exclusion criteria were refined during full-text review for clarity and specificity to the review’s objectives following discussion among the research team. The PRISMA Extension for Scoping Reviews (PRISMA-ScR) checklist was referenced to ensure systematic reporting of this scoping review [22].

### Identification of the Research Question

Overall, we were interested in mapping the existing intervention literature for stress reduction or stress management in stroke survivors. As an emerging and inconsistently defined field, we anticipated enormous heterogeneity in the way stress is defined, operationalized, and measured across studies, and in the way that stress and stress prevention studies are designed. Based on the conceptual framework presented in Fig. 1, we included any intervention studies which measured stress or a related concept as an outcome; therefore, the included studies encompassed both interventions specifically designed for stress reduction, and interventions that did not explicitly aim to reduce stress, but which reported a reduction in a stress-related outcome, such as self-reported stress. The following broad aims, which attempt to capture this heterogeneity of studies under the Lazarus and Folkman framework [20], guided this scoping review:

1. To map the range of interventions trialed addressing stress management in stroke survivors and to identify which interventions are potentially efficacious for reducing stress, or increasing resilience and coping skills.
2. To identify the average duration of study length and follow-up. Stroke is a chronic condition with recovery occurring for months and years after the initial event. Further, it would be of interest to assess the potential impact of interventions on longer-term outcomes that may be affected by acute improvements in stress management. Ideally, stress intervention models would match the natural history of stroke and stress-related problems, and this would be reflected in follow-up times of adequate duration.
3. To map the multidimensional range of outcome measures that have been used for stress, resilience, and coping in stroke survivors. We anticipated heterogeneous literature, with no broadly accepted outcome measure for psychological stress. Acute and chronic stress can be quantified using various approaches including self-reported, psychometric assessments, as well as physiological biomarkers.
4. To identify whether early intervention for stress translates into a reduction in longer-term stress-related clinical outcomes as shown in Fig. 1, including depression, anxiety, and PTSD. Although we do not expect psychological stress to be the sole cause of mental disorders poststroke, most cases of depression and anxiety can, to some extent, be traced back to the influence of exogenous or endogenous stressors [27]. Therefore, although an improvement in stress management or coping, or a reduction in perceived stress will not explain all the variation in poststroke depression or anxiety, we would expect a positive effect on mood disorders due to a preventive effect. However, since stress management does not specifically treat mood symptoms, significant changes in mood, and diagnosis with stress-related disorders, may not be observed.
5. The success of any intervention is in part dependent on the successful implementation within its environment and context. Therefore, we collected information on whether studies considered implementation outcomes, including potential barriers and limitations, feasibility and acceptability, and economic considerations. This is particularly relevant for stress management approaches, as they may be time consuming, expensive, and difficult to scale up to larger populations.

### Search and Screening Methods

The search strategy was designed around the aims of the review and included two key concepts, stroke and stress. The search was designed to align with the stress, appraisal, and coping framework used to conceptualize stress in this review. Broadly, interventions for stress reduction were operationalized in the following way: any intervention (pharmacological or nonpharmacological) delivered in individual, family, or group settings, incorporating strategies to prevent or delay the development of excessive stress, promote coping strategies or resilience, to improve optimism and wellbeing, or to improve or relieve stress-related outcomes, including symptoms of mood disturbance. We included all types of intervention studies (randomized controlled trials [RCTs] and quasi-experimental designs). The research question and corresponding search strategy are defined using the Population, Intervention, Comparator, Outcome, Study design (PICOS) framework (see Supplementary File 1). A complete description of the strategies for database searching, filtering methods, abstract identification, and screening was provided in a previously published protocol [23]. The search for this scoping review was iterative in nature. It began with a gold standard set of articles that informed the selection of medical subject headings (MeSH), keywords, and keyword phrases. The strategy was further refined through reference checking and forward and backward citation checking. Search strategies from reviews in relevant areas were also searched. The search strategy drew on the work of the Cochrane Stroke Group to operationalize search terms for stroke. The search strategy was developed in Medline before being optimized for Embase, resulting in the need to include some additional Emtree terms to capture additional relevant citations. The strategy was then translated to PsycInfo, CINAHL, and the Cochrane Library. All databases were searched from inception until March 11, 2019, and updated on December 12, 2019. The search was updated again prior to submission on September 1, 2020 (Supplementary File 2). Database searches were restricted to subjects and English language citations only. Primary evidence (empirical research) only was included.

An assessment of study quality is optional in scoping reviews; however, may be conducted to gain an appreciation of the quality of evidence in a field. We did not exclude articles based on quality or design; however, we did conduct an assessment of study quality using the Cochrane Risk of Bias tool or the Mixed Methods Appraisal Tool (MMAT), where appropriate, in order to evaluate the existing quality of evidence in the area.

### Identifying Relevant Studies

The search yield was imported into Covidence software and duplicates were removed. Title and abstract, and full-text screening were completed separately by members of the research team (M.H., M.I., P.G., M.K., and KC), with each article independently screened by two team members. Discrepancies during screening and reviewing were resolved by a consensus among all reviewers. Inconsistencies were discussed and resolved, and inclusion criteria were refined to improve the application of inclusion/exclusion criteria.

To be included, studies had to meet the following criteria: (a) include an intervention; (b) involve human adult stroke survivors (age ≥18 years); (c) be written in English; and (d) include at least one outcome measure related to stress or resilience. Outcome measures were consistent with the Lazarus and Folkman [20] stress–coping–appraisal framework, and incorporated changes in direct measures including perceived stress, resiliency, coping skills, and problem-solving, as well as measures of changes in state stress and aligned constructs, including emotional distress, coping, resilience, QoL, and symptoms of anxiety or depression. Although some of the latter measures do not measure stress directly, they were included as outcomes in several publications consistent with the Lazarus and Folkman framework [20] which aimed to improve coping or problem-solving skills, were hypothesized to thereby improve QoL, and reduce emotional distress in stroke survivors [28–31], and were therefore included in this review. This reflects the substantial impact that stress can have on physical and mental health. Exclusion criteria included: (a) nonexperimental (e.g., observational, case–control, cross-sectional, longitudinal) studies (i.e., without implementation of an intervention); and (b) relevant reviews (systematic and meta-analysis), but reference lists were hand-searched to identify additional eligible articles. Studies could be randomized or nonrandomized (quasi-experimental).

### Charting the Data

Data extraction was independently completed by five reviewers (M.H., M.I., P.G., M.K., and K.C.). The data extraction spreadsheet was designed to capture all relevant details required to answer the research questions and included: author, year published, country, sample size, and population characteristics were recorded (e.g., age, stroke type, and time since stroke, severity measures, comorbidities), outcome measures associated with stress, length of follow-up, type of intervention, duration of intervention, control group, any measures of acceptability and feasibility, any measures of barriers, study limitations, and any measures of cost or cost-effectiveness. The spreadsheet was refined via an iterative process in collaboration with all reviewers.

### Collating, Summarizing, and Reporting the Results

We tabulated key information from included studies descriptively. We categorized interventions by intervention type, study duration, and follow-up (in line with aims 1 and 2), explored how stress and stress-related disorders were measured in these studies (in line with aims 3 and 4), and assessed effectiveness (in line with aim 1) and other measures that may affect implementation, including barriers, acceptability, and cost-effectiveness (in line with aim 5). Records in the PsycInfo database receive a classification code, which is used to categorize the document according to the primary subject matter. We used these classification codes to map interventions to higher-order keywords to categorize them. Findings were presented in a narrative synthesis.

### Deviations From the Protocol

We originally stated that we would identify potential findings which may help to inform practice and/or guidelines. Some recent guidelines for CVD identify stress as an important risk factor [32]; however, there are no best-practice recommendations for the management or reduction of stress in stroke survivors. The included studies were overwhelmingly early phase and/or feasibility trials, and as such no recommendation for an approach to stress management could be determined based on the evidence synthesis included in this review.

### Assessment of Study Quality

Quality appraisal was used to broadly assess the quality of the literature, to determine where the field currently lies in terms of evidence development. It was not intended to stratify papers into a hierarchy of evidence, and publications were not excluded from the review based on quality. To assess the quality of the quantitative studies, the Cochrane Risk of Bias 2 (RoB2) quality appraisal tool was used [33]. For any included qualitative or mixed-method studies, the MMAT was used [34]. The MMAT does not have an overall rating category, and therefore we used the following guide to assess the overall risk of bias associated with each publication: (a) strong (80% or more of the quality indicators were met), (b) moderate (between 40% and 80% of the quality indicators were met), and (c) weak (less than 40% of the quality indicators were met).

## Results

### Included and Excluded Articles

**Fig. 2.**
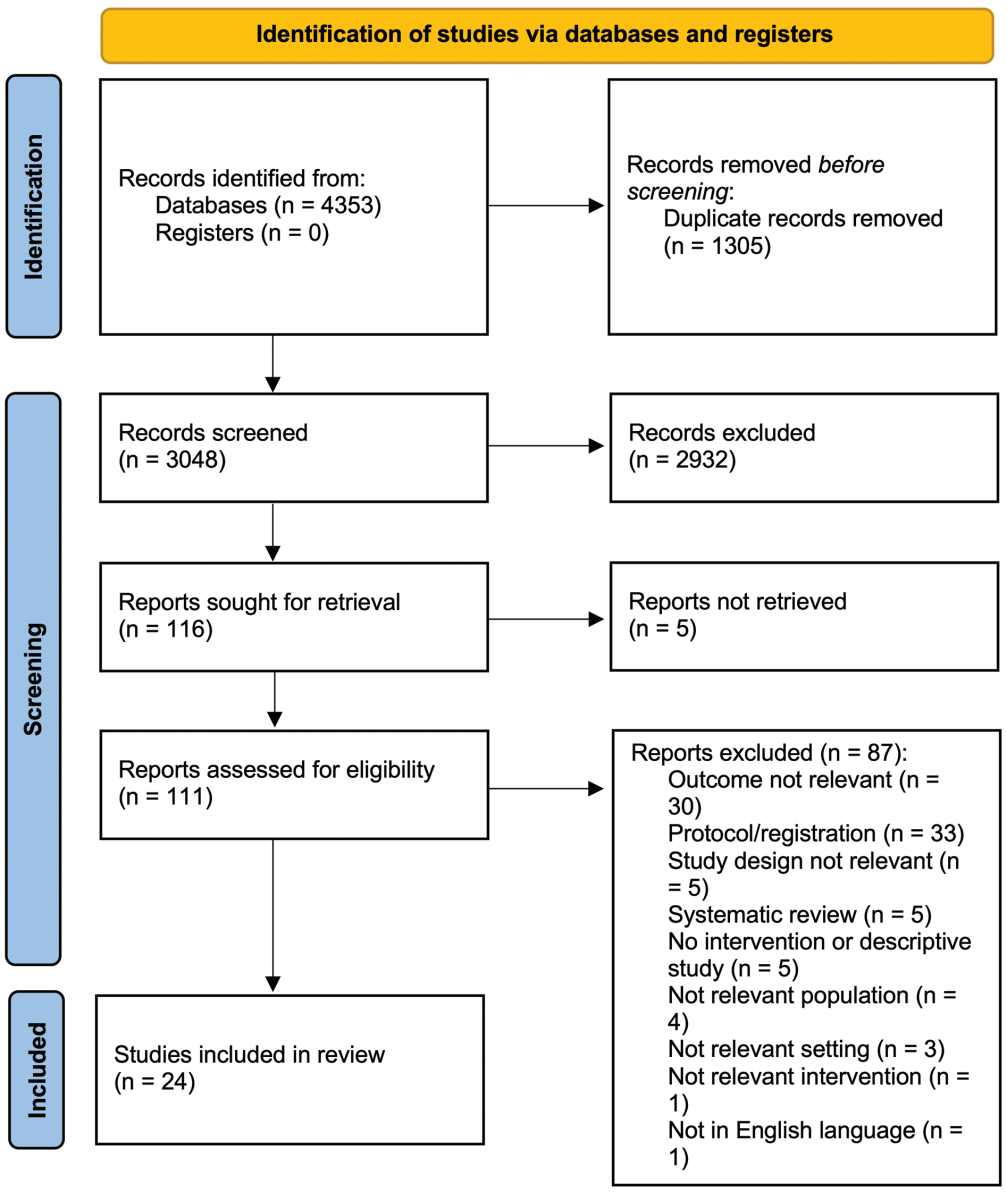
PRISMA flow diagram [35].

The study selection process is summarized in the PRISMA flow diagram (Fig. 2) [35]. The initial search, which was conducted on March 11, 2019, identified 2,653 references after deduplication. The search was updated on December 12, 2019, and again prior to submission on September 1, 2020, after which there was a total of 3,140 studies imported to Covidence, with 3,048 available for screening following deduplication. Of these, 116 articles were considered potentially relevant after initial exclusions of titles and abstracts. A further 92 were excluded after a two-person review of the full text. A total of 24 articles were included in this review [28–31, 36–55].

### Study Characteristics

Study characteristics, including country, study design, participants, intervention, comparator, and length of follow-up, are summarized in Supplementary File 3. Included studies were published between 1992 and 2020, with most (*n* = 17; 70%) published in the last 5 years [28, 31, 36, 38–42, 45–48, 51–55].

There was significant variability in study design, including RCTs and non-RCTs, and uncontrolled before and after studies. The type, duration, and frequency of intervention used also varied across studies, with interventions running from five sessions over 1 week (aromatherapy foot bath and massage) [46], to 6 months (antidepressant treatment with sertraline [49]; home-based psychoeducational program [50]). Most interventions ran weekly sessions over approximately 8–12 weeks (*n* = 15) [29–31, 40–42, 44, 45, 48–52, 54, 55]. The duration of follow-up also varied significantly between studies, ranging from 0 (immediate follow-up) to 12 months. Finally, as we placed no restrictions on study type beyond intervention studies, three studies used qualitative thematic analyses (via survey or interview) [37, 40, 48], in which stress emerged as a theme.

Whilst the reported sample sizes varied from 8 to 166 participants, most of the included studies had relatively small sample sizes, with 16 out of 24 studies recruiting fewer than 50 participants [28, 29, 36, 37, 39–42, 44–48, 51–54].

The populations in the included studies varied in terms of time poststroke, and whether a broad or selected population was recruited. Most studies reported time postonset of stroke; only Nour et al. [29] and Chouliara and Lincoln [40] did not explicitly define time poststroke; Nour et al. [29] stated that participants had finished active rehabilitation. We categorized the populations according to the critical time points of stroke recovery proposed in Bernhardt et al. [56], including hyperacute (0–24 hr), acute (1–7 days), subacute (7 days to 6 months), and chronic (>6 months) phases. The majority of studies (*n* = 15) [28, 30, 31, 41, 43–48, 50–54] involved participants recruited in the chronic phase of recovery. Five studies involved participants recruited during the subacute phase of stroke [37, 38, 42, 49, 55]. Two studies recruited participants during the acute period, whilst patients were hospitalized [36, 39]. We based our assessments on the mean or median times poststroke reported in the studies; therefore, a small number of studies may have included participants across multiple phases of recovery, primarily subacute to chronic. Studies rarely specified whether included participants were first or recurrent stroke survivors or details of stroke type.

Three of the included studies used a selected population (i.e., those reporting high distress, or those with an existing diagnosis of depression, anxiety, or fatigue [41, 49, 52]), in order to assess the effect of an intervention on these outcomes. Many studies excluded people with a progressive neurological disorder or cognitive dysfunction, reduced life expectancy, subdural hematomas, moderate or severe aphasia, or who partook in excessive drinking or drug abuse [31, 38, 39]. Studies typically justified this approach by stating that collecting and reliably interpreting data from these patients can present significant challenges.

### Study Interventions

The types of interventions trialed for stress management across the included studies are summarized in Table 1.

**Table 1.** Characteristics of interventions used to address stress levels in stroke survivors

Type	Subtype	Theory/hypothesis	Studies
Psychosocial			
Social support networks	Social support	Social support intervention would improve the support experienced by stroke survivors, as such leading to better psychosocial outcome.	Friedland and McColl (1992) [43]
Cognitive processes	MBSR	MBSR will reduce mental fatigue after stroke and TBI.	Johansson et al. (2012) [44]
	Problem-solving therapy	Problem-solving therapy will improve coping strategy, QoL, and reduce emotional distress in stroke survivors.	Visser et al. (2016) [31] Chalmers et al. (2019) [28]
	Meditation	Meditation will cultivate mindfulness to train ttention and awareness, to achieve a mentally clear and emotionally calm and stable state.	Love et al. (2020) [47]
Rehabilitation/ neuropsychological rehabilitation	Memory rehabilitation	Memory rehabilitation will develop patients’ ability to cope with or compensate for residual memory deficits, as well as promoting participation.	Chouliara and Lincoln (2016) [40]
	Leisure rehabilitation	Leisure education will improve QoL and depression in stroke survivors.	Nour et al. (2002) [29]
Behavioral	Proactive coping intervention	Stroke survivors with better proactive coping skills will experience improved self-efficacy and QoL.	Tielemans et al. (2015) [30]
Motor processes	Physical activity program/exercise	Improving and maintaining physical activity levels will improve the overall health, including psychological functioning, cognitive functioning, and sleep, of adults with acquired brain injury.	Jones et al. (2016) [45] Colledge et al. (2017) [41]
	Aquatic therapy	Aquatic therapy will minimize anxiety, fatigue, and depression, which tend to be barriers to stroke rehabilitation.	Perez-de la Cruz (2020) [51]
Psychotherapy	Positive psychotherapy	Positive psychotherapy will alleviate psychological distress after acquired brain injury.	Cullen et al. (2018) [42] Terrill et al. (2018) [54]
	SFBT	SFBT will reduce depression and anxiety symptoms, generate a constructive attitude, and increase self-efficacy in patients poststroke.	Wichowicz et al. (2017) [55]
Creative arts therapy	Person-centered arts program	Participation in an arts program will improve the emotional and mental wellbeing of stroke survivors.	Baumann et al. (2013) [37]
Treatment	cCBT	cCBT will alleviate emotional distress and mental health problems such as anxiety and depression after experiencing a stroke.	Simblett et al. (2017) [52]
	PosMT	Training in positivity using the PosMT audio tool could be added to rehabilitation for prevention or management of poststroke psychological problems.	Mavaddat et al. (2017) [48]
Multicomponent	Psychoeducation (mailed and home visit)	Home-based psychoeducation will improve the perceived health of stroke survivors by decreasing depression, fatigue, and the negative impact of stroke.	Ostwald et al. (2014) [50] Stubberud et al. (2019) [53]
	Promoting psychosocial wellbeing following stroke	Applying a dialog-based intervention drawing on narrative theory, supported conversation for people with aphasia, and guided self-determination will promote a sense of coherence in life and reduce threats to wellbeing after stroke, such as feelings of chaos and lack of control.	Bragstad et al. (2020) [38]
Training	Skills-based intervention informed by CBT, DBT, and trauma-informed care	Training in areas including cognitive restructuring/reappraisals, adaptive thinking, mindfulness, distress tolerance, impact of the illness/injury, understanding triggers, and role and identity changes will prevent chronic emotional distress in stroke survivors and their caregivers.	Bannon et al. (2020) [36]
	Biofeedback training	HRV biofeedback will improve autonomic dysfunction, cognitive impairment, and psychological distress.	Chang et al. (2020) [39]
Alternative medicine			
Alternative medicine	Aromatherapy (foot bath and massage)	Back massage and foot bath using essential oils will improve stress, body temperature, mood state, and fatigue levels of stroke patients.	Lee et al. (2017) [46]
Pharmacological			
Antidepressant drugs	Sertraline (50–100 mg daily)	Sertraline, a SSRI, will have positive effects on both depressive symptoms and other relevant poststroke domains including emotional distress and QoL.	Murray et al. (2005) [49]

*cCBT* computerized cognitive behavior therapy; *DBT* dialectical behavior therapy; *HRV* heart rate variability; *MBSR* mindfulness-based stress reduction; *PosMT* positive mental training; *QoL* quality of life; *SFBT* solution-focused brief therapy; *SSRI* selective serotonin reuptake inhibitor; *TBI* traumatic brain injury.

We used the PsycInfo database to map each intervention to its broader subject heading category and identified the theory or hypothesis associated with each that would lead to an improvement in stress-related outcomes. Although there was wide variability in the types of intervention trialed, most (22; 92%) utilized psychosocial interventions targeted at the individual level. These include social support [43], cognitive processes including mindfulness-based stress reduction, meditation and problem-solving therapy [28, 31, 44, 47], rehabilitation or neuropsychological rehabilitation targeted at memory or leisure [29, 40], a behavioral proactive coping intervention [30], physical activity programs [41, 45, 51], psychotherapy including both positive psychotherapy [42, 54] and solution-focused brief therapy [55], creative arts therapy [37], cognitive behavioral therapy or positive mental training [48, 52], multicomponent interventions consisting of home-based visits and mailed information, based on principles of psychoeducation [38, 50, 53] and training modules based on developing skills in either cognitive behavior therapy/cognitive reappraisal or heart rate variability biofeedback [36, 39]. The remaining two studies assessed alternative medicine (aromatherapy massage and foot bath [46]), and pharmacological treatment (the selective serotonin reuptake inhibitor antidepressant sertraline [49]). There were no organizational-level interventions in the included studies.

### Stress Outcome Metrics

Table 2 summarizes how stress or stress-relevant outcomes were measured in the included studies. Not all studies were designed to examine stress or resilience as a primary outcome, and as such the included outcomes were not necessarily primary outcomes. Studies were included into this scoping review only if the stress was specifically discussed in the results section of the study. This may include data from qualitative interviews or surveys, or within a subscale of another measure (e.g., QoL).

**Table 2.** Characteristics of outcome measures used to assess interventions for stress measurement

Outcome type	Outcome measurement scale	Studies
Stress	10-item Perceived Stress Scale (PSS-10)	Colledge et al. (2017) [41] Ostwald et al. (2014) [50]
	Depression Anxiety Stress Scales (DASS-21)	Cullen et al. (2018) [42]
	General Health Questionnaire (GHQ)	Friedland and McColl (1992) [43]
	Kessler Psychological Distress Scale (K10)	Jones et al. (2016) [45]
	Mental Fatigue Scale (MFS)	Johansson et al. (2012) [44]
	Social Readjustment Rating Scale (SRRS)	Lee et al. (2017) [46]
	Emotional Distress Scale (EDS)	Murray et al. (2005) [49]
Coping	Utrecht Proactive Coping Competence scale (UPCC)	Tielemans et al. (2015) [30]
	Coping Inventory for Stressful Situations	Visser et al. (2016) [31]
Problem-solving	Social Problem-Solving Inventory-Revised (SPSIR)	Chalmers et al. (2019) [28] Visser et al. (2016) [31]
Resilience	10-item Connor Davidson Resilience Scale (CD-RISC)	Terrill et al. (2018) [54] Perez-de la Cruz (2020) [51]
	Brief Resilience Scale (BRS)	Love et al. (2020) [47]
Depression/anxiety/mood	Center for Epidemiologic Studies Depression Scale (CES-D)	Chalmers et al. (2019) [28] Visser et al. (2016) [31] Love et al. (2020) [47]
	Beck Depression Inventory (BDI)	Colledge et al. (2017) [41] Nour et al. (2002) [29] Simblett et al. (2017) [52]
	Beck Anxiety Inventory(BAI)	Simblett et al. (2017) [52]
	Hospital Anxiety and Depression Scale (HADS)	Chalmers et al. (2019) [28] Stubberud et al. (2019) [53] Tielemans et al. (2015) [30] Wichowicz et al. (2017) [55]
	Comprehensive Psychopathological Rating Scale (CPRS)	Johansson et al. (2012) [44]
	Multiple Affective Adjective Checklist (MAACL)	Lee et al. (2017) [46]
	Montgomery–Åsberg Depression Rating Scale (MADRS)	Murray et al. (2005) [49]
	Presence of emotionalism (increased tearfulness and pathologic crying was recorded as a dichotomous variable)	Murray et al. (2005) [49]
	Geriatric Depression Scale (GDS)	Ostwald et al. (2014) [50]
	PROMIS-Depression Short Form 8b	Terrill et al. (2018) [54]
	State-Trait Anxiety Inventory (STAI-Y)	Love et al. (2020) [47]
Quality of life	Stroke Specific Quality of Life Scale (SS-QOL)	Chalmers et al. (2019) [28] Tielemans et al. (2015) [30] Visser et al. (2016) [31]
	Global subjective rating of change in quality of life (QoL) was measured according to a validated visual analog scale	Murray et al. (2005) [49]
	Sickness Impact Profile	Nour et al. (2002) [29] Friedland and McColl (1992) [43]
	Older People’s Quality of Life Questionnaire (OPQOL)	Terrill et al. (2018) [54]
	Short From 36 Health Survey (SF-36)	Perez-de la Cruz (2020) [51]
	EuroQol EQ-5D-5L	Visser et al. (2016) [31]
Life satisfaction	Satisfaction with Life Scale (SWLS)	Colledge et al. (2017) [41]
	Authentic Happiness Inventory (AHI)	Cullen et al. (2018) [42]
	Leisure Satisfaction Scale	Nour et al. (2002) [29]
	Likert scales: current life satisfaction, and the difference from prestroke life satisfaction	Cullen et al. (2018) [42]
Qualitative analysis	Open-ended descriptive survey	Chalmers et al. (2019) [28]
	Semi-structured interviews	Chouliara and Lincoln (2016) [40] Mavaddat et al. (2017) [48] Baumann et al. (2013) [37]

Several different psychometric scales were used to assess stress or related constructs; however, we found no studies assessing stress biomarkers. In our search strategy, we included terms for coping and resilience, resulting in the inclusion of studies that measured stress, and resilience, coping, problem-solving, stress-related disorders (including anxiety, depression, and PTSD), life satisfaction, and QoL measures, where stress was reported as a subcomponent of the measure. Of the 14 studies which included a psychometric measurement of stress, coping, or resilience, 8 reported using a stress-specific outcome measure [41–46, 49, 50], with others recording stress-related constructs via a coping scale [30, 31], problem-solving scale [28, 31], or resilience scale [47, 51, 54].

Most studies measured stress-related disorders via a scale for symptoms of depression, anxiety, or mood (*n* = 16) [28–31, 36, 39, 41, 44, 46, 47, 49, 50, 52–55]. The Center for Epidemiologic Studies Depression Scale (CES-D) [28, 31, 47], Beck Depression Inventory (BDI) [29, 41, 52], and the Hospital Anxiety and Depression Scale (HADS) [28, 30, 36, 39, 53, 55] were the most commonly used measures.

Ten studies assessed QoL and/or life satisfaction [28–31, 41–43, 49, 51, 54]; some studies reported a stress measure as a subscale of this. For example, Friedland and McColl [43] used the General Health Questionnaire (GHQ) and the Sickness Impact Profile (SIP) to measure “psychosocial adjustment”. As mentioned above three qualitative thematic analysis studies [37, 40, 48] were included which did not measure stress outcomes but which highlighted stress as an emerging theme. For example, Baumann et al. [37], a descriptive study of an art therapy program aiming to reduce distress during rehabilitation, did not explicitly measure stress but described individual participants’ experiences of distress associated with stroke.

### Effectiveness and Implementation Outcomes

For each intervention, we assessed the effectiveness in terms of both reduction in stress or related construct, and reduction in stress-related mental health disorders (anxiety, depression, or PTSD); implementation measures including barriers and limitations, feasibility and acceptability, and any cost analysis or cost-effectiveness, were reported. Collectively, these features are likely to inform the further development of an intervention for eventual use in practice (Table 3).

**Table 3.** Measures of effectiveness, acceptability, feasibility, and cost-effectiveness

Study	Stress reduction	Reduction in anxiety or mood disorder	Barriers and limitations	Feasibility and acceptability	Cost-effectiveness
Bannon et al. (2020) [36]	Increased scores on resiliency variables, including self-efficacy, mindfulness, and perceived coping in Recovering Together training dyads, but not control dyads from baseline to post-test.	Participation in Recovering Together was associated with baseline to post-test decrease in symptoms of depression, anxiety, and PTS in stroke survivors and caregivers.	Small feasibility trial Patients discharged before they could be approached Low internal consistency on measures with reversed scored items for patients at baseline	Clinical staff not invested in project Low recruitment Treatment satisfaction was high Adherence and acceptability of procedures was high	NR
Baumann et al. (2013) [37]	Weekly art sessions during rehabilitation. Participants reported that the sessions offered a source of relaxation, tranquility or calmness(qualitative analysis).	NR	No long-term follow-up (1 week after final session) Small sample size (*n* = 18)	All but one patient indicated a wish to continue arts activities in the future	NR
Bragstad et al. (2020) [38]	No between-group differences in psychosocial wellbeing at 12 months poststroke.	No statistically significant between-group difference in depression, sense of coherence, or health-related QoL at 12 months.	The intervention was designed both to be delivered uniformly and to be individualized. The competing aims may have compromised session delivery Sample may not be representative; informed consent was difficult to obtain in the stroke unit	Composite adherence score showed that 117 (80.1%) of the intervention trajectories satisfied the criteria for high-fidelity intervention adherence Participants reported finding the intervention helpful	NR
Chalmers et al. (2019) [28]	Problem-solving therapy did not produce a significant change in overall problem-solving (SPISR; proxy measure for emotional distress).	Slight reduction after therapy on the CES-D and no reduction on the HADS-A.	No randomization Use of waitlist control group No controlling for confounding Problems with recruitment Small sample size (*n* = 28)	Participants generally reported that each therapy session was helpful and enjoyable	NR
Chang et al. (2020) [39]	Average HR decreased compared with baseline in the HRVBF, but no significant difference between groups.	HADS score significantly decreased in the HRVBF group at 1 and 3 months but not in the control group.	Small sample size Short follow-up time Did not log self-practice during follow-up	5/40 patients dropped out after randomization Study reported that the intervention was feasible, but it was not clear on what basis this was reported	NR
Chouliara and Lincoln (2016) [40]	Memory rehabilitation led to perceived benefits in participants’ ability to effectively manage stress (qualitative analysis).	NR	Small sample size (*n* = 20) Qualitative analysis did not directly assess some aspects Separate results not reported for stroke survivors only	NR	NR
Colledge et al. (2017) [41]	No effect of exercise training on perceived stress.	Descriptive reduction in depressive symptoms at follow-up.	Small sample size (*n* = 32) Exploratory trial (descriptive analysis only) Selection bias No nonintervention control group	NR	NR
Cullen et al. (2018) [42]	Mean difference of −5.8 points on the DASS-21 Stress scale (intervention vs. controls) at week 20 after positive psychology intervention.	Mean difference of −9.6 points on the DASS-21 Anxiety scale (intervention vs. controls) at week 20 after positive psychology intervention.	Small sample size (*n* = 37) Exploratory trial Not designed or powered for efficacy	63% retention (15 completers) Authors considered intervention feasible to deliver and acceptable to participants	NR
Friedland and McColl (1992) [43]	No differences between social support intervention and control groups on psychosocial variables including the GHQ.	NR	Relatively high attrition rate Timing of intervention	NR	NR
Johansson et al. (2012) [44]	Statistically significant reduction in reported mental fatigue, including reduction in sensitivity to stress item, following MBSR compared with baseline and controls.	Significantly decreased scores for depression and anxiety on the CPRS after 8 weeks of MBSR.	Small sample size (*n* = 29) Some participants who discontinued found the program time consuming, or difficult to travel to attend appointments	NR	NR
Jones et al. (2016) [45]	A statistically significant reduction in psychological distress (K10 scale) of 2.76 points immediately after the myMoves program (*p* = .001).	NR	Small sample size (*n* = 24) Contact with study participants occurred outside of the intervention program Mixed population (stroke and TBI)	Participants completed an average of 5.6/6 sessions Participants reported a high level of overall satisfaction with the program (95.7%) The program required little clinician contact time, with an average of 32.8 min per participant over 8 weeks	NR
Lee et al. (2017) [46]	Statistically significant reduction in the social readjustment rating scale (means not reported).	NR	Small sample size (*n* = 14) Poor reporting of study methodology and results	NR	NR
Love et al. (2020) [47]	Small, but nonsignificant, postmeditation increase in resilience observed.	NR	Small sample size (*n* = 35) No control group Selection bias Sampling in single stroke clinic Exclusion of participants lost to follow-up	NR	NR
Mavaddat et al. (2017) [48]	In qualitative interviews, stroke survivors reported benefits of the positive mental training program in handling stress, improved mood, and coping ability.	Four stroke survivors had improved scores on PANAS; two stroke survivors had improved scores, and one stroke survivor had a worse score on the HADS.	Small sample size (*n* = 10) Self-selected sample Not all participants completed the full 12-week program	7/10 stroke survivors reported positive benefits from listening and would recommend to others Participants with moderate aphasia found it difficult to concentrate and did not persist with the study	£38 for access to the full audio program (in 2013 GBP).
Murray et al. (2005) [49]	No statistically significant effect of sertraline treatment on EDS score, however there was a reduction from baseline in both groups regardless of treatment. Some improvement in QoL and emotionalism.	The MADRS score decreased substantially in both treatment groups, with no significant differences between them at 6 and 26 weeks.	High discontinuation rate (39% in the treatment and 49% in the placebo group) Selection of patients with minor depression only	NR	NR
Nour et al. (2002) [29]	At post-test, the experimental group (leisure rehabilitation) obtained statistically significantly better scores for total, psychological, and physical QoL, although effect sizes were small.	No statistically significant difference between groups for depression (BDI).	Small sample size Extra time (on average 20 min per session) provided to intervention group Some participants did not complete the program	Participants reported satisfaction with leisure activities following the intervention	NR
Ostwald et al. (2014) [50]	No effect of mailed or home-based psychoeducational intervention on stress as measured by the PSS-10.	No effect of mailed or home-based psychoeducational intervention on depression scores (GDS).	Sample size not large enough for subgroup analyses The sample is not representative—included only those over 50 years of age who were being discharged home with a spouse Analysis of multiple outcomes in this study possibly increased the type I error rate.	84% of the dyads completed the study 12-month follow-up. Dyads that did not complete the study were older, had higher caregiver support scores and spent more days in inpatient rehabilitation than those who finished the study	Number, length, and content of each contact was tracked, allowing for analysis of costs. An average of two home visits a month during the initial 6 months at home postdischarge from inpatient rehabilitation could be delivered at a mean cost of $2,500 per dyad.
Perez-de la Cruz (2020) [51]	In the experimental group, significant differences from baseline were found in the resilience variables (*p* < .001) and these improvements were maintained 1 month after completing the treatment program.	NR	Small sample size (*n* = 41) Short follow-up (1 month)	All the participants completed all the sessions and complied with the proposed program	NR
Simblett et al. (2017) [52]	All groups demonstrated a decrease in symptoms of distress, measured via the BDI and BAI, and the NEADL across time associated with computerized CBT and computerized cognitive remediation therapy.	Trend toward reduced depression scores on the BDI for computerized CBT and computerized cognitive remediation therapy. Smaller trend for anxiety symptoms.	Small sample size (*n* = 28) Feasibility and acceptability were the primary outcomes for this study, not efficacy No blinding to intervention of outcome assessors	Feasibility and acceptability were the primary outcomes for this study. Recruitment rate for the intervention ran below the expected rate Broadly feasible, although some aspects required more flexibility Majority suggested intervention was useful, relevant and easy to use	Not explicitly measured. However, the idea of small groups was primarily introduced as a means of improving the feasibility of delivery by reducing costs associated with providing psychological therapy.
Stubberud et al. (2019) [53]	No change in self-efficacy after training in metacognitive strategies for improving attention, problem-solving, fatigue management, adaptive coping responses, and the use of CBT techniques as measured using the GSE scale.	Decrease in overall score on the HADS, driven by a change in the anxiety subscale. No change in the depression subscale.	Small sample size (*n* = 8, of which 5 are stroke survivors) Results not presented separately for stroke and TBI survivors Self-reported outcomes only No control group	All subjects completed the interventions	NR
Terrill et al. (2018) [54]	Study not designed to measure effect of intervention—the data collected in this study were used to identify feasibility of a positive psychology app. 8/10 dyads still used positive psychology in their everyday lives at follow-up. Measured CD-RISC, but results not reported.	Measured PROMIS-Depression Short Form 8b, but results not reported.	Small sample size; 11 stroke survivor/carer dyads. One dyad discontinued, final 10 dyads	Participants reported satisfaction with the intervention Stroke survivors were fatigued by training session One of the dyads dropped out of the study Remaining dyads engaged in a mean of 4.08 individual and 3.62 couple activities per week	Not explicitly reported; referenced a Cochrane review stating that use of apps to administer self-management programs was cost-effective.
Tielemans et al. (2015) [30]	No effect of self-management intervention compared with an education intervention in coping skills on the UPCC scale.	Trend favoring the self-management intervention on the HADS.	Study did not include more severely affected stroke survivors Self-assessment used to assess outcomes Outcome measures too generic to detect changes Study too small to detect outcome differences in partners (*n* = 57 partners) Sample selected by hospital staff Usual care was not controlled	Of 58 patients assigned to the self-management intervention, 56 started the intervention and 46 attended at least three quarters of the intervention sessions	NR
Visser et al. (2016) [31]	Improvement in coping strategy as measured by the Coping Inventory for Stressful Situations between groups (problem-solving therapy vs. control).	Depression score did not differ significantly between the groups over time (CES-D).	No active control groups Intervention was investigated within outpatient rehabilitation and may not be generalizable to other settings Sample reported a relatively high utility score compared with other stroke populations	The low dropout rate and positive feedback suggest that an open group design is feasible and effective in outpatient stroke rehabilitation	NR
Wichowicz et al. (2017) [55]	Increased self-efficacy and constructive attitudes in the SFBT group compared with controls (Mini-Mental Adjustment to Cancer and Self-efficacy Scale).	Reduced anxiety and depression scores in the SFBT group compared with controls (HADS).	62 completers (100 randomized) Participants relatively fit and may not be representative 35 withdrawals from study Lengthy procedure Lack of complete randomization	Potential SFBT participants who refused to participate may have found the procedure of psychotherapy too lengthy	NR

*BAI* Beck Anxiety Inventory; *BDI* Beck Depression Inventory; *CD-RISC* 10-item Connor Davidson Resilience Scale; *CES-D* Center for Epidemiologic Studies Depression Scale; *CPRS* Comprehensive Psychopathological Rating scale; *DASS-21* Depression Anxiety Stress Scales; *EDS* Emotional Distress Scale; *GBP* British pounds; *GDS* Geriatric Depression Scale; *GHQ* General Health Questionnaire; *GSE* General Self-Efficacy; *HADS* Hospital Anxiety and Depression Scale; *HR* heart rate; *HRVBF* heart rate variability biofeedback; *MADRS* Montgomery–Åsberg Depression Rating Scale; *MBSR* mindfulness-based stress reduction; *NEADL* Nottingham Extended Activities of Daily Living; *NR* not reported; *PANAS* Positive and Negative Affect Schedule; *PROMIS* Patient-Reported Outcomes Measurement Information System; *PSS-10* Perceived Stress Scale; *PTS* post-traumatic stress; *QoL* quality of life; *SFBT* solution-focused brief therapy; *SPISR* Social Problem-Solving Inventory-revised; *TBI* traumatic brain injury; *UPCC* Utrecht Proactive Coping Competence.

In order to address our research question “to identify which interventions are potentially efficacious for reducing stress or increasing resilience and coping” we compiled a descriptive overview of the reported effectiveness of the intervention in each study. Positive effects on stress, resilience, coping, or psychological QoL/life satisfaction were reported in 13 of the 24 included studies [29, 31, 36, 37, 40, 42, 44–46, 48, 51, 52, 55]. Baumann et al. [37] and Chouliara and Lincoln [40] reported qualitative reductions in stress associated with an inpatient art program and memory rehabilitation, respectively. The other studies reported a quantitative improvement in stress-related outcomes associated with a number of intervention types: skills-based training based on principles of cognitive behavior therapy [36], positive psychology [42, 48], mindfulness-based stress reduction [44], physical activity program [45], aromatherapy massage, and footbath [46], leisure rehabilitation [29], aquatic therapy [51], computerized cognitive behavior therapy or cognitive remediation [52], problem-solving therapy [31], and solution-focused brief therapy [55]. Whilst this represents many included studies (54%), most of these had relatively small sample sizes, ranging from 14 to 166, with 10/13 studies recruiting fewer than 30 stroke survivors. Most of these were reported as exploratory, quasi-experimental, or feasibility studies, and reported other methodological concerns including lack of active control group and significant dropout rates. Some of these results were also not numerically reported or reported as a qualitative perceived reduction only.

The impact of the interventions on longer-term stress-related problems, primarily symptoms of depression or anxiety, were considered in 75% (18/24) included studies. Of these, seven reported a quantitative decrease in these symptoms [36, 39, 42, 44, 47, 53, 55].

In addition to effectiveness results, we also collated implementation outcomes reported across studies, primarily acceptability and feasibility, in order to assess whether the potential long-term sustainability of intervention had been considered in the included studies. Where studies included an explicit measure of feasibility or acceptability from participants, feedback was generally positive [28, 29, 31, 36–39, 42, 45, 46, 48, 50–52, 54]. Several studies reported low dropout rates and high adherence to the intervention strategy (*n* = 8) [31, 36, 38, 45, 50, 51, 53, 54]. Broadly, this suggests a willingness to participate in these intervention studies. However, several studies did report problems with recruitment or other barriers to participation, including potential refusal to participate based on the time commitments or participation burden required for some interventions (*n* = 8) [30, 36, 42–44, 50, 54, 55]. Further, some interventions reported differential dropout rates for different groups of participants, particularly those with aphasia (*n* = 2) [30, 48].

We also investigated whether studies reported some measure of cost or cost-effectiveness analysis. This is also an important consideration for upscaling and eventually implementing a novel intervention in practice. Only four of the included studies referred to or measured the costs of the intervention. Mavaddat et al. [48] and Ostwald et al. [50] both reported the costs of providing the intervention per participant. In Terrill et al. [54] and Simblett et al. [52], although costs were not explicitly measured, both reported that the intervention was expected to be cost-effective based on its features. The cost-effectiveness of any intervention was not reported.

### Assessment of Study Quality

We used the Cochrane Risk of Bias tool to assess intervention studies, and the MMAT to assess mixed-method and qualitative studies (Supplementary File 4). The included quantitative studies (*n* = 21) [28–31, 36, 38, 39, 41–47, 49–55] had methodological limitations, including unclear recruitment techniques, small sample sizes, high attrition rates, failure to control for important confounders, use of nonvalidated measures and inconsistent reporting. Studies scored most strongly (low risk of bias) on sequence generation (12/21), allocation concealment (11/21), incomplete outcome data (18/21), and selective outcome reporting (16/21). The categories where studies overall were weaker (associated with a high or unclear risk of bias) included blinding of participants and personnel (16/21), blinding of outcome assessors (14/21), and other sources of bias (20/21). Quality scoring of the qualitative or mixed-methods papers (*n* = 3) [37, 40, 48] suggested that these papers had a moderate risk of bias. Studies were not excluded based on quality. The assessment of quality for each of the included studies is presented in Supplementary Appendix.

## Discussion

This scoping review is the first to map the breadth of research that has been conducted around stress interventions for stroke survivors. Here, we have mapped the types and effectiveness of the trialed interventions, as well as the outcome measures used to assess stress or related constructs. We also considered aspects of implementation reported in the studies, including patient acceptability, feasibility, limitations, and cost-effectiveness. We identified a total of 24 studies that recruited mixed populations of stroke survivors in terms of susceptibility to stress-related outcomes and time poststroke and identified a variety of primarily psychosocial interventions delivered to individuals to directly address stress management or promote related constructs such as resilience, problem-solving, or coping. Although all studies reported the effect of the intervention on stress or stress-related outcome measures, a correlation to recovery outcomes was not consistently addressed or investigated across studies. We also identified significant methodological issues associated with most studies, and a tendency for studies to be at an early or feasibility stage. Furthermore, even though the majority of included studies appeared to be feasibility or exploratory trials, our search did not identify any larger subsequent or follow-up studies to this early work. Overall, despite the trend toward positive outcomes, the limitations of the included studies made it difficult to conclusively identify the most effective interventions. Despite these shortcomings, we found that the number of relevant publications increased over time, suggesting that stress and stress management are progressively being considered important for stroke survivors.

Psychological distress is commonly reported following stroke and is associated with a number of significant cognitive and psychological problems, but based on this review, the evidence base for psychotherapeutic interventions is small and equivocal. Any recommendation for an intervention designed to manage excessive stress, with the ultimate aim of preventing stress-related disorders and improving recovery, should be evidence based in order to justify the allocation of resources, and in order to reduce harms from potentially ineffective interventions. Although there is emerging evidence for targeting stress for the prevention of some disorders such as CVD, at present the relevance of intervening on stress in stroke populations remains unclear [57, 58]. Broadly, the population with the greatest benefit:risk ratio is not defined, the potential range of interventions remains broad, and there is no agreement on the best outcome measures to use for stress. The studies included here also tended to emphasize treatment rather than prevention of emotional distress. This is in agreement with two recent systematic reviews of dyadic interventions for caregivers and stroke survivors, which focused on interventions to reduce stress in caregivers. The results of these studies highlighted substantial limitations of interventions for carers and stroke survivors including weighting toward treating rather than preventing stress, and a lack of customizable interventions which would allow tailoring to account for the heterogeneity of stroke survivors’ needs [59, 60].

### Stress Management Interventions and Their Delivery After Stroke

A wide variety of interventions were trialed in the included studies, which were predominantly small in scale and based on very localized or intensive solutions generally run for an 8–12-week period. Often considerations on how such an intervention might scale up to the broader population were not included/made. Several studies reported poor adherence and/or recruitment due to the burden of participation. Stress management interventions that require large amounts of time and travel may lead to adherence problems in stroke survivors and should be an important consideration in the development of any intervention. Unlike pharmaceuticals, funding mechanisms for integrating new psychosocial interventions into health systems are less clear. Further, funding for sufficiently large effectiveness trials and eventual reimbursement should also be considered. These factors were rarely considered in the included studies. Adequate resourcing, and considering the burden of participation for stroke survivors and health systems, are important considerations in the design of any preventive intervention. This is likely to be magnified for an issue such as stress management, which will require significant adherence to achieve its goals.

In order to address issues around implementation, scalability, and cost-effectiveness, it may be worth considering interventions that have been trialed in other populations that may be likely associated with smaller participant burden, lower costs, and high effectiveness. For example, mobile technologies and mHealth interventions might be a low-cost, simple method of delivering modular, customized mental health support to mitigate stress in stroke survivors. Mobile interventions based on cognitive training approaches have been used successfully for stress-related cognitive problems such as memory impairment [61]. However, to date, there is very little regulation of these interventions and systematic research on the potential benefits of mHealth interventions for stress management is not currently available.

### Target Population

The relevant population for targeting stress management to prevent the stress-related sequelae of stroke also remains unclear. Prevention of stress after stroke could be either universal (i.e., offered to all individuals poststroke), or targeted to a higher risk population of stroke survivors identified as having elevated levels of stress prior to the stroke, or those most at risk of developing adverse stress-related outcomes. The studies included in this review were mixed; some included a broader population of stroke survivors (21/24), whilst others included only those at higher risk of stress and related sequelae, such as emotional distress or symptoms of depression (3/24). It is also unclear at which phase of stroke recovery is best to intervene. Although most of the included studies were conducted during the chronic phase of stroke recovery (i.e., >6 months poststroke), at present it remains unknown as to when might be the optimum time to manage stress throughout the recovery trajectory. Whilst the relative risk of negative health outcomes due to stress may be small compared with other risk factors, a recent review highlighted the importance of tackling stress in people with high baseline cardiovascular risk, such as stroke survivors, because this translates into a larger difference in absolute risk [62]. In order to determine when and in which population stress management might be most clinically and cost-effective, additional studies are required which monitor stress and related sequelae over time.

Additionally, several studies excluded people with a progressive neurological disorder, cognitive dysfunction, or aphasia. Studies typically justified this approach by stating that collecting and reliably interpreting data from these patients can present significant challenges. However, a high proportion of stroke patients have cognitive or speech, and language impairments, and if studies of interventions aiming to reduce psychological distress among stroke patients fail to involve such patients, the findings may not be representative of the wider stroke population [37]. Therefore, a number of these studies with specific inclusion/exclusion criteria may not be generalizable to the broader population with stroke and have limited external validity.

### Measurement of Stress

There was a lack of consistency in measuring stress between studies. Significant uncertainty in the field of stress research more broadly lies in the use of standardized techniques or tools to measure the level of stress. Whilst stress has been linked to an increased risk of secondary stroke, CVD, and psychopathology in stroke survivors, and has a negative impact on the trajectory of recovery from stroke, the best approach to measure stress in research and clinical settings remains unclear [63, 64]. Compared with other known behavioral risk factors for chronic diseases, such as smoking, nutrition, and physical activity, psychosocial constructs such as stress are difficult to define objectively [65]. The response to stress can be measured using self-report, or a physiological measurement. A number of scales have been developed for the measurement of stress. The Perceived Stress Scale (PSS-10) [66], Symptom Checklist 90 (SCL-90) questionnaire [67], and the Depression Anxiety Stress Scales (DASS-21) [68] tend to be the most widely used validated tools found in the literature for the subjective assessment of stress. In this review, we found considerable variation across studies in terms of self-reported approaches for measuring stress. Several studies did not use any stress measurement tool, instead of reporting subscale results of a QoL scale or similar to infer emotional distress, qualitative results, or used an unstandardized tool, making it difficult to compare study results. Related constructs such as resilience also appeared infrequently in our search, with only three studies reporting the results of an intervention on a resilience scale.

Similarly, no studies reported changes in any biomarkers for stress. There are numerous biological pathways linking stress to disease outcomes, and as a result, a number of physiological stress measures or biomarkers are commonly used in stress research, primarily based on stress-related changes in neuroendocrine signaling [69]. One of the major alternatives to the psychometric assessment of stress is to assess levels of stress hormones, in blood or saliva [7]. A number of studies have considered a measurement of blood cortisol levels; however, these measures also suffer from serious limitations in terms of their accuracy because stress is not the only factor that evokes changes in the levels of these hormones [7, 63]. Stress hormones measured within the saliva and blood can change quickly in their concentration and fluctuate significantly over time [70]. These issues mean that single-time point analyses using blood or saliva may be noninformative. Of course, it is possible to collect multiple samples across time, but the practical considerations and participant burden mean this is typically not feasible. One recent study has used an objective measure of the stress level in the form of hair cortisol, to associate stroke outcome with stress [71]. The hair sample for the determination of cortisol level instead of blood, saliva, or urine offers several advantages in stroke–stress research. Firstly, its analysis can provide an accurate assessment of the long-term integrated level of cortisol over the course of the months. Secondly, its measurement is not affected by acute stress variables and is not subject to diurnal variation, and finally, baseline hair samples obtained within the week after stroke provides information about stress and cortisol level prior to the stroke incident. The relatively stable and noninvasive nature of hair cortisol as a stress biomarker may make this an ideal marker in future studies of stress poststroke.

### Does Stress Management Prevent Stress-Related Disorders?

Finally, it has not yet been shown that a change on a subjective stress scale is a proxy for patient-relevant outcomes, such as prevention of anxiety or depression. Based on the included studies, there is only limited evidence to suggest that the included interventions to manage stress and related constructs will translate into a reduction in stress-related disorders in stroke survivors. In the included studies, only 7 of the 18 that considered symptoms of stress-related disorders reported a decrease in symptoms associated with the intervention. This may not be unexpected, since these programs were designed to address stress management and not treat existing symptoms of anxiety or depression. Whilst improved stress management is likely to be an important precursor to effective prevention of depression or anxiety, it remains unclear where best to target these efforts. In future studies, it will be important to establish not only consistent and relevant measures of stress, but also the link between an improvement in stress management or stress levels and reductions in the risk of known stress-associated disorders such as depression, anxiety, CVD, and mild cognitive impairment. Further, studies should examine longitudinally the impact of early compared with late stress mitigation, to examine whether stroke survivors who receive these preventive measures at varying time periods in recovery are less likely to develop stress-related problems.

### Limitations

There are a number of potential limitations to this review. While we conducted a comprehensive search using key databases and hand searching, it is possible that the review may have missed some relevant studies. We also included only papers in English and did not conduct a search of the gray literature. There may be evidence of program impacts in the evaluation and other technical reports, not available here.

## Conclusion

The successful management of chronic stress in stroke survivors is likely to improve psychological and cognitive recovery outcomes and improve rehabilitation contacts. However, most intervention studies we identified were small, primarily consisting of feasibility studies to inform larger trials. There was a trend toward a positive effect of stress management interventions in stroke survivors, in terms of reduction in perceived stress levels, and a smaller trend toward a reduction in symptoms of anxiety and depression associated with improved stress management. It is unclear at this stage which population would benefit most, and which intervention could be recommended based on effectiveness and implementation outcomes. Additionally, it is still unclear how best to measure and monitor stress in stroke survivors. In order for the field to address the gaps and inconsistencies identified here, we must be able to effectively assess stress levels in individuals over time.

Overall, despite the heterogeneity of the studies covered in this review, there was a clear trend toward positive outcomes, and the number of studies conducted is increasing over time. As a field, this area is gaining attention, and future well-designed and properly powered studies will provide important answers to the questions raised in this review. Given that an increasing number of studies have identified that stress represents an unmet need for stroke survivors and is an important contributor to increased risk of poor recovery outcomes, addressing stress provides an opportunity to improve long-term outcomes for stroke survivors.

## Supplementary Material

kaac002_suppl_Supplementary_File_1

kaac002_suppl_Supplementary_File_2

kaac002_suppl_Supplementary_File_3

kaac002_suppl_Supplementary_File_4
