# Supplementary material for: Psychological Stress Management and Stress Reduction Strategies for Stroke Survivors: A Scoping Review
Source: Ann Behav Med. 2022 Jun 11;57(2):111–30. doi: 10.1093/abm/kaac002 (PMC9899067; doi:10.1093/abm/kaac002)
Supplement: kaac002_suppl_Supplementary_File_1 [file kaac002_suppl_supplementary_file_1.docx]

**Supplementary File 1: Translation of the research question into a bibliographic search strategy**

|  | **Research question** | **Search strategy** |
| --- | --- | --- |
| Population | Stroke survivors | Stroke terms informed by those utilised in reviews by the Cochrane stroke group |
| Intervention | Any intervention designed to reduce stress or stress-related outcomes | Stress terms developed based on a gold standard set of articles, other stroke reviews, and forward and backward citation searching |
| Comparator | Any | - |
| Outcome | Short- and long-term outcomes of stress, including perceived stress, stress biomarkers, and stress-related disorders | Identified during title and abstract, or full-text searching |
| Study design | Any intervention study | A methodological filter for intervention studies |
