# Supplementary material for: Psychological Stress Management and Stress Reduction Strategies for Stroke Survivors: A Scoping Review
Source: Ann Behav Med. 2022 Jun 11;57(2):111–30. doi: 10.1093/abm/kaac002 (PMC9899067; doi:10.1093/abm/kaac002)
Supplement: kaac002_suppl_Supplementary_File_2 [file kaac002_suppl_supplementary_file_2.docx]

**Supplementary File 2:** Search strategies

**Table 1:** MEDLINE search strategy (1946 to September, 2020)

| **Line** | **Search terms** | **N hits** |
| --- | --- | --- |
| 1 | cerebrovascular disorders/ or exp basal ganglia cerebrovascular disease/ or exp brain ischemia/ or exp carotid artery diseases/ or exp intracranial arterial diseases/ or exp intracranial arteriovenous malformations/ or exp "intracranial embolism and thrombosis"/ or exp intracranial hemorrhages/ or stroke/ or exp brain infarction/ or vasospasm, intracranial/ or vertebral artery dissection/ | 353056 |
| 2 | (stroke or poststroke or post-stroke or cerebrovasc* or brain vasc* or cerebral vasc* or cva* or apoplex* or SAH).ti,ab,kw,kf. | 312452 |
| 3 | ((brain* or cerebr$ or cerebell* or intracran* or intracerebral) adj5 (isch?emi* or infarct* or thrombo* or emboli* or occlus*)).ti,ab,kw,kf. | 111748 |
| 4 | ((brain* or cerebr$ or cerebell* or intracran* or intracerebral) adj5 (h?emorrhage* or h?ematoma* or bleed*)).ti,ab,kw,kf. | 54095 |
| 5 | hemiplegia/ or exp paresis/ or gait disorders, neurologic/ | 25590 |
| 6 | (hemipleg$ or hemipar$ or pareis or paretic).ti,ab,kw,kf. | 26880 |
| 7 | exp stroke/ and survivors/ | 1309 |
| 8 | Stroke rehabilitation/ | 13373 |
| 9 | 1 or 2 or 3 or 4 or 5 or 6 or 7 or 8 | 564665 |
| 10 | Stress, Psychological/ or Adaptation, psychological/ | 197673 |
| 11 | Resilience, Psychological/ or resilien*.ti,ab,kw,kf. | 35078 |
| 12 | Optimism/px or (optimis* or positivity or bounce back).ti,ab,kw,kf. | 111940 |
| 13 | ((stress* or distress) adj2 (acute or mild or chronic or perceived or self-perceived or psychological or psychosocial or mental or physiologic* or emotional or posttraumatic or post-traumatic)).ti,ab,kw,kf. | 142858 |
| 14 | (stress adj1 (manage* or reduc* or minimi*)).ti,ab,kw,kf. | 14584 |
| 15 | ((coping or cope) adj1 (abilit* or style*)).ti,ab,kw,kf. | 5134 |
| 16 | 10 or 11 or 12 or 13 or 14 or 15 | 444707 |
| 17 | 9 and 16 | 5472 |
| 18 | randomized controlled trial.pt. | 511962 |
| 19 | controlled clinical trial.pt. | 93820 |
| 20 | Random allocation.sh. | 103476 |
| 21 | Double blind method/ or single blind method/ | 187536 |
| 22 | clinical trial.pt. | 524467 |
| 23 | Placebos/ | 35050 |
| 24 | Comparative study/ or Evaluation study/ | 2064386 |
| 25 | Evaluation studies.pt. | 26 |
| 26 | ((singl* or doubl* or tripl* or trebl*) adj1 (blind* or mask*)).ti,ab. | 174421 |
| 27 | (placebo* or random* or trial* or groups).ti,ab. | 3466298 |
| 28 | intervention*.ti,ab. | 1004003 |
| 29 | 18 or 19 or 20 or 21 or 22 or 23 or 24 or 25 or 26 or 27 or 28 | 5918963 |
| 30 | 17 and 29 | 2072 |
| 31 | exp animals/ not humans.sh. | 4729286 |
| 32 | 30 not 31 | 1924 |
| 33 | limit 32 to english language | 1806 |

**Table 2:** EMBASE search strategy (1974 to September 2020)

| **Line** | **Search terms** | **N hits** |
| --- | --- | --- |
| 1 | cerebrovascular disease/ or basal ganglion hemorrhage/ or cerebral artery disease/ or cerebrovascular accident/ or stroke/ or exp carotid artery disease/ or exp brain hematoma/ or exp brain hemorrhage/ or exp brain infarction/ or exp brain ischemia/ or exp cerebrovascular malformation/ or exp intracranial aneurysm/ or exp occlusive cerebrovascular disease/ | (740673) |
| 2 | (stroke or poststroke or post-stroke or cerebrovasc* or brain vasc* or cerebral vasc* or cva* or apoplex* or SAH).ti,ab,kw. | 500081 |
| 3 | ((brain* or cerebr$ or cerebell* or intracran* or intracerebral) adj5 (isch?emi* or infarct* or thrombo* or emboli* or occlus*)).ti,ab,kw. | 164723 |
| 4 | ((brain* or cerebr$ or cerebell* or intracran* or intracerebral) adj5 (h?emorrhage* or h?ematoma* or bleed*)).ti,ab,kw. | 83224 |
| 5 | hemiparesis/ or hemiplegia/ or paresis/ | 57325 |
| 6 | (hemipleg$ or hemipar$ or pareis or paretic).ti,ab,kw. | 42525 |
| 7 | stroke survivor/ | 1460 |
| 8 | 8 stroke rehabilitation/ | 3612 |
| 9 | 1 or 2 or 3 or 4 or 5 or 6 or 7 or 8 | 935261 |
| 10 | mental stress/ or post-stroke depression/ | 86729 |
| 11 | coping behavior/ or stress management/ | 63871 |
| 12 | psychological resilience/ | 3762 |
| 13 | resilien*.ti,ab,kw. | 40097 |
| 14 | optimism/ | 5093 |
| 15 | (optimism or optimistic or positivity or bounce back).ti,ab,kw. | 120226 |
| 16 | ((stress* or distress) adj2 (acute or mild or chronic or perceived or self-perceived or psychological or psychosocial or mental or physiologic* or emotional or posttraumatic or post-traumatic)).ti,ab,kw. | 191899 |
| 17 | (stress adj1 (manag* or reduc* or minimi*)).ti,ab,kw. | 20060 |
| 18 | ((coping or cope) adj1 (abilit* or style*)).ti,ab,kw. | 6898 |
| 19 | 10 or 11 or 12 or 13 or 14 or 15 or 16 or 17 or 18 | 460273 |
| 20 | 9 and 19 | 8675 |
| 21 | crossover-procedure/ or double-blind procedure/ or randomized controlled trial/ or single-blind procedure/ or (random* or factorial* or crossover* or cross over* or placebo* or (doubl* adj blind*) or (singl* adj blind*) or assign* or allocat* or volunteer* or trial* or groups).ti,ab. | 5431957 |
| 22 | 20 and 21 | 2128 |
| 23 | (animal/ or nonhuman/) not human/ | 6154995 |
| 24 | 22 not 23 | 1948 |
| 25 | limit 24 to english language | 1871 |
| 26 | limit 25 to (books or chapter or conference abstract or conference paper or "conference review" or editorial or letter or note) | 672 |
| 27 | 25 not 26 | 1199 |
| 28 | limit 27 to dd=20191205-20200901 | 26 |

**Table 3:** PsycInfo search strategy (1967 to September 2020)

| **Line** | **Search terms** | **N hits** |
| --- | --- | --- |
| 1 | exp cerebrovascular disorders/ | 28249 |
| 2 | (stroke or poststroke ot post-stroke or cerebrovasc* or brain vasc* or cerebral vasc* or cva* or apoplex* or SAH).ti,ab,id. | 39838 |
| 3 | ((brain* or cereb* or cerebell* or intracran* or intracerebral) adj5 (isch?emi* or infarct* or thrombo* or emboli* or occlus*)).ti,ab,id. | 10075 |
| 4 | ((brain* or cereb* or cerebell* or intracran* or intracerebral) adj5 (h?emorrhage* or h?ematoma* or bleed*)).ti,ab,id. | 3866 |
| 5 | hemiplegia/ | 1060 |
| 6 | general paresis/ | 219 |
| 7 | (hemipleg* or hemipar* or paresis or paretic).ti,ab,id | 6177 |
| 8 | 1 or 2 or 3 or 4 or 5 or 6 or 7 | 52105 |
| 9 | exp psychological stress/ | 8918 |
| 10 | emotional adjustment/ or coping behavior/ | 62724 |
| 11 | optimism/ | 4251 |
| 12 | (optimis* or positivity or positivism or bounc* back).ti,ab,id. | 25843 |
| 13 | ((stress* or distress) adj2 (acute or mild or chronic or perceived or self-perceived or psychological or psychosocial or mental or physiologic* or emotional or posttraumatic or post-traumatic)).ti,ab,id. | 105135 |
| 14 | (stress adj1 (manag* or reduc* or minimi*)).ti,ab,id. | 12183 |
| 15 | ((coping or cope) adj1 (abilit* or style*)).ti,ab,id. | 8486 |
| 16 | 9 or 10 or 11 or 12 or 13 or 14 or 15 | 196996 |
| 17 | 8 and 16 | 1162 |
| 18 | exp intervention/ | 107424 |
| 19 | clinical trials/ | 11740 |
| 20 | treatment effectiveness evaluation/ | 24698 |
| 21 | (placebo* or random* or intervention*).ti,ab,id. | 561331 |
| 22 | 18 or 19 or 20 or 21 | 586799 |
| 23 | 17 and 22 | 257 |
| 24 | limit 23 to (chapter or "column/opinion" or "comment/reply" or dissertation or editorial or letter) | 55 |
| 25 | 23 not 24 | 202 |
| 26 | limit 25 to english language | 181 |
| 27 | limit 26 to up=20191205-20200901 | 13 |
