# Supplementary material for: Psychological Stress Management and Stress Reduction Strategies for Stroke Survivors: A Scoping Review
Source: Ann Behav Med. 2022 Jun 11;57(2):111–30. doi: 10.1093/abm/kaac002 (PMC9899067; doi:10.1093/abm/kaac002)
Supplement: kaac002_suppl_Supplementary_File_3 [file kaac002_suppl_supplementary_file_3.docx]

**Supplementary File 3:** Characteristics of included studies (N=24) evaluating interventions to address stress levels in stroke survivors.

| **Study ID** | **Country** | **Study design** | **N** | **Participants** | **Intervention** | **Control** | **Follow-up** |
| --- | --- | --- | --- | --- | --- | --- | --- |
| Bannon *et al.* (2020)[36] | US | RCT | 17 stroke survivors, 17 caregivers | Stroke survivors newly admitted to a Neurosciences Intensive Care Unit (randomised within 1 week of hospitalisation) | Recovering Together skills based dyadic intervention. Seven modules presented over 6 sessions (2 at bedside, 4 after discharge via video platform). | Minimally enhanced usual care control (care as usual with provision of informational pamphlet on stroke and stroke recovery) | 3 months |
| Baumann *et al*. (2013)[37] | UK | UBA | 18 | Inpatient stroke survivors, average length of stay 110 days who were medically stable and likely to be in hospital for at least two weeks to attend sessions. | Art program consisting of 4-6 one-on-one art sessions facilitated by various artists. | No control | NR |
| Bragstad *et al*. (2020)[38] | Norway | RCT | 322 | Stroke survivors within 4 weeks of acute stroke, medically stable with enough cognitive function to participate | Dialogue-based intervention to promote psychosocial wellbeing. Eight 60-90 min sessions over 17 weeks delivered in the community (primarily the patient’s home). | Standard care control group | 12 months |
| Chalmers *et al*. (2019)[28] | New Zealand | N-RCT | 29 | Young stroke survivors (18-65 years) who had experienced a stroke within the previous 6 months to 3 years and who felt their QoL had been affected as a result of the stroke. | Problem solving therapy. Six group sessions run over 6 weeks (4–8 participants per group). The first two sessions lasted for 90 min and the last four lasted for 60 min. | Waitlist control  (no intervention) | 3 months |
| Chang *et al*. (2020)[39] | Taiwan | RCT | 40 | Stroke survivors within 1 week of their first-ever ischemic stroke. | HRV biofeedback training. Four sessions over 4 days, with fortnightly reminder phone calls during follow up (5 calls total). | Usual care control group | 3 months |
| Chouliara & Lincoln (2016)[40] | UK | RCT | 20 | Participants with memory impairment following TBI, stroke and MS. Of the total population, 5 reported previous stroke. | Group-based memory rehabilitation. One initial individual session followed by 10 weekly group sessions of 1.5h each. | No control | 7 months after randomisation |
| Colledge *et al*. (2017)[41] | Switzerland | RCT | 48 | 15 aSAH survivors, 16 meningioma patients and 17 healthy controls, mean 44 months after neurosurgical or endovascular intervention. | Moderate aerobic exercise training 3-5 times per week for 12 weeks. | Matched meningioma patients and healthy controls | 6 months |
| Cullen *et al*. (2018)[42] | Scotland | RCT | 27 | Stroke survivors median 5.7 months post-stroke recruited from stroke outpatient clinics and a stroke psychology service outpatient waiting list, with presence of emotional distress (moderate or above on at least one subscale of the DASS-21), medically stable. | Brief positive psychotherapy intervention, delivered via 8 individual weekly outpatient sessions by one research psychologist. | Usual care within clinical service | 12 weeks |
| Friedland & McColl (1992)[43] | US | RCT | 88 | Stroke survivors average 11.4 months post-stroke (23% <6 months post-stroke). Subjects were excluded if had a history of psychiatric admission or been on anti-depressant medication. | Social support intervention - 6 group sessions, with up to 6 additional sessions provided as required. | Usual care | 3 months |
| Johansson *et al*. (2012)[44] | Sweden | RCT | 29 | Stroke or TBI survivors, ranging from 3.3 years since TBI/stroke in the MBSR group (n=15) to 9.8 years in the control group (n=14), aged 30-65 years. Recovered from neurological symptoms but reporting significant mental fatigue. | MBSR consisting of 8 weekly 2.5h long sessions, one day-long silent led retreat, and guided home practice of 45 min 6 days per week. | Waitlist controls | No follow-up (immediate assessment) |
| Jones *et al*. (2016)[45] | Australia | UBA | 24 | Acquired brain injury population. Included 20 stroke survivors and 4 TBI survivors approximately 4.4 years post-event. | myMoves physical activity program comprising 6 modules delivered over 8 weeks via e-mail, plus weekly contact with physical therapist via e-mail and phone. | Within-group baseline measures | 3 months |
| Lee *et al*.  (2017)[46] | South Korea | RCT | 14 | Stroke survivors approximately 6 months to 2 years post-stroke. | Aromatherapy back massage (30 min) and foot bath (30 min) five times over one week, in addition to general physical therapy. | General physical therapy only | No follow-up (immediate assessment) |
| Love *et al*. (2020)[47] | US | RCT | 35 | Stroke survivors (mean age = 58.3 ± 13.9) who have experienced stroke within the last 12 months (mean of 112.5 ± 103.4 days post stroke). Ischemic stroke n=26, haemorrhagic stroke n=5, and transient stroke n=10. | Breath-based meditation for 4 weeks. One meditation session per week with an expert and meditation daily at home on other days. | No control group | No follow-up (immediate assessment) |
| Mavaddat *et al*. (2017)[48] | UK | UBA | 15 | Stroke survivors ranging from 1-10 years post stroke (n=10) and carers (n=5) from stroke support groups. | PosMT audio program (12 audio tracks of 18 min each, to be listened to every day for one week with a different track each week for 12 weeks). | No control group | No follow-up (immediate assessment) |
| Murray *et al*. (2005)[49] | Sweden | RCT | 123 | Stroke survivors (mean of 128 ± 97 days post-stroke) included if fulfilled DSM-IV criteria of major depressive episode (n=76) or minor depressive disorder (n=47). | Sertraline (50–100 mg/day) over 26 weeks. | Placebo | 6 weeks (interim);  26 weeks |
| Nour *et al*.  (2002)[29] | Canada | RCT | 14 | Stroke survivors finished active rehabilitation, no communication problems or any major cognitive deficit that would interfere with completion of questionnaires or the program. | Home leisure educational program. 12-step program delivered in 10 weekly hour-long sessions at home, tailored to each participant. | A flexible "social" program consisting of weekly hour–long home visits with conversations on different topics. | No follow-up (immediate assessment) |
| Ostwald *et al.* (2014)[50] | US | RCT | 159 | 159 stroke survivors and caregiver dyads (318 total participants). One member of the dyad had a diagnosis of stroke within the previous 12 months and needed assistance with activities of daily living. | Home-based psychoeducational and monthly mailed information programs for stroke-caregiving dyads. Average of 16 visits of 70 min each dyad over a 6-month period. | Mailed information only | 12 months |
| Pérez-de la Cruz (2020)[51] | Spain | RCT | 41 | 41 stroke patients, 15 received physiotherapy on dry land (mean age = 62.7 ± 13.4 years; time after stroke 5.2 ± 2.7 years), 13 received aquatic therapy (mean age = 63.8 ± 13.6 years; time after stroke; 5.1 ± 4.2 years); and 13 received both aquatic therapy and dry land physiotherapy mean age = 61.4-63.8 years, mean time post-stroke = 5.1-5.6 years. | Aquatic therapy performed twice a week for 12 weeks (a total of 24 sessions). Each session was 45 min long and consisted of an initial warm-up, lasting 10 min. | Dry land physiotherapy | 1 month |
| Simblett *et al*. (2017)[52] | UK | RCT | 28 | Approximately 1-year post-stroke (0.89-1.19 years). No neurodegenerative condition, high score on BDI and BAI. | cCBT in addition to usual care. Eight modules of approx. 1h each over 8 consecutive weeks in small groups in community settings. | Active comparison (computerised training exercises to rehearse cognitive skills) in addition to usual care. | 3 months |
| Stubberud *et al*. (2019)[53] | Norway | UBA | 8 | 5 stroke survivors, 3 TBI; mean 40 months post-injury recruited from a cognitive rehabilitation unit, with post-acquired brain injury fatigue (FSS score >5). | Multifaceted intervention including 3 modules over 12 days in 1 month, covering lifestyle factors and adaptive coping strategies (5 days), goal management training (5 days) and emotional regulation (2 days). The subjects received approximately 3h of intervention each day, totalling 36h of in-patient intervention. | Baseline control | 9 months |
| Terrill *et al*. (2018)[54] | US | RCT | 11 stroke survivors, 11 partners | Stroke survivors mean 2.9 years post-stroke, one or both partner(s) had to report depressive symptoms. | Dyadic positive psychology-based intervention consisting of one brief face-to-face training session and an 8-week self-administered intervention (participants were instructed to engage in at least two activities alone and two together each week). | Waitlist controls | 3 months |
| Tielemans *et al.* (2015)[30] | The Netherlands | RCT | 113 stroke survivors, 57 partners | Stroke survivors mean 18.8 months post-stroke, with participation problems in vocational, social or leisure domains by endorsing at least 2 items on the Restriction scale of the USER-Participation. | Proactive coping self-management intervention (‘Plan Ahead!’). 7 group sessions over 10 weeks, 6 × 2h sessions in the first 6 weeks and 1 × 2h booster session in week 10. | Education intervention | 3 and 9 months |
| Visser *et al*. (2016)[31] | Belgium and The Netherlands | RCT | 166 | Stroke survivors median 7.3 months post-stroke. | Problem-solving therapy added to the last 8 weeks of outpatient rehabilitation. Eight group sessions of 1.5h each. | Standard outpatient rehabilitation only consisting of pre-defined modules including physiotherapy, occupational therapy, psychology, speech therapy, and social work. | 6 and 12 months |
| Wichowicz *et al*. (2017)[55] | Poland | RCT | 100 | First ever ischemic stroke survivors 14 days after discharge (approx. 3 weeks post-stroke). | SFBT, ten sessions (60 min per session) over 8-10 weeks. | No psychotherapy/usual care | No follow up (immediate assessment) |

Abbreviations: RCT, randomized controlled trial; UBA, uncontrolled before and after study; NR, not reported; N-RCT, non-randomized controlled trial; QoL, quality of life; HRV, heart rate variability; TBI, traumatic brain injury; MS, multiple sclerosis; aSAH, aneurysmal subarachnoid haemorrhage; DASS-21, depression, anxiety and stress Scale - 21 Items; MBSR, mindfulness-based stress reduction; PosMT, positive mental training; DSM-IV, diagnostic and statistical manual of mental disorders, fourth edition; BDI, Beck depression inventory; BAI, Beck anxiety inventory; cCBT, computerised cognitive behavioural therapy; FSS, fatigue severity scale; USER-Participation, Utrecht scale for evaluation of rehabilitation-participation; SFBT, solution-focused brief therapy.
