# Supplementary material for: Psychological Stress Management and Stress Reduction Strategies for Stroke Survivors: A Scoping Review
Source: Ann Behav Med. 2022 Jun 11;57(2):111–30. doi: 10.1093/abm/kaac002 (PMC9899067; doi:10.1093/abm/kaac002)
Supplement: kaac002_suppl_Supplementary_File_4 [file kaac002_suppl_supplementary_file_4.docx]

**Supplementary File 4:** Quality assessment of included studies

**Table 1:** Quality scores for the quantitative studies included in this review

| **No** | **Study** | **Sequence generation** | **Allocation concealment** | **Blinding of participants and personnel** | **Blinding of outcome assessors** | **Incomplete outcome data** | **Selective outcome reporting** | **Other sources of bias** |
| --- | --- | --- | --- | --- | --- | --- | --- | --- |
| 1 | Bannon *et al*. (2020)^28^ | Low | Low | High | High | Low | Low | High |
| 2 | Bragstad *et al.* (2020)^30^ | Low | Low | High | Low | Low | Low | High |
| 3 | Chalmers *et al*. (2017)^31^ | High | High | High | High | Low | Low | High |
| 4 | Chang *et al*. (2020)^32^ | Low | Low | High | Low | Low | Low | High |
| 5 | Colledge *et al*. (2017)^34^ | High | High | High | High | Low | Low | High |
| 6 | Cullen *et al*. (2018)^35^ | Low | Low | Low | Low | Low | Low | Unclear |
| 7 | Friedland & McCall (1992)^36^ | Low | High | High | High | Low | Low | Unclear |
| 8 | Johansson *et al*. (2012)^37^ | Unclear | Unclear | High | High | Low | Low | Unclear |
| 9 | Jones *et al*. (2016)^38^ | High | High | High | High | Low | High | Unclear |
| 10 | Lee *et al*. (2017)^39^ | High | High | High | High | High | Unclear | High |
| 11 | Love *et al*. (2020)^40^ | High | High | High | High | High | High | High |
| 12 | Murray *et al*. (2005)^42^ | Low | Low | Low | High | Low | Low | High |
| 13 | Nour *et al*. (2002)^43^ | High | High | High | High | Low | Low | High |
| 14 | Ostwald *et al*. (2014)^44^ | Low | Low | High | High | Low | Low | High |
| 15 | Pérez-de la Cruz (2020)^45^ | Low | Low | High | Low | Low | Low | Low |
| 16 | Simblett et al. (2017)^46^ | Low | Low | Low | High | Low | Low | High |
| 17 | Stubberud *et al*. (2017)^47^ | High | High | High | High | Low | Low | High |
| 18 | Terrill *et al*. (2018)^48^ | Low | High | High | Low | High | High | High |
| 19 | Tielemans *et al*. (2015)^49^ | Low | Low | Low | Unclear | Low | Low | High |
| 20 | Visser *et al*. (2016)^50^ | Low | Low | Low | Low | Low | Low | Unclear |
| 21 | Wichowicz *et al*. (2017)^51^ | High | Low | High | Low | Low | High | High |

**Table 2:** Quality scores for the qualitative studies included in this review

| No. | Study |  | Quality Score |
| --- | --- | --- | --- |
| 1 | Baumann *et al*. (2013)^29^ (qualitative interview) |  | Moderate |
| 3 | Chouliara & Lincoln (2016)^33^ (mixed methods) |  | Moderate |
| 4 | Mavaddat *et al*. (2017)^41^ (qualitative interview and questionnaire) |  | Moderate |
